# Identification of immune subtypes of cervical squamous cell carcinoma predicting prognosis and immunotherapy responses

**DOI:** 10.1186/s12967-021-02894-3

**Published:** 2021-05-24

**Authors:** Yimin Li, Shun Lu, Shubin Wang, Xinhao Peng, Jinyi Lang

**Affiliations:** 1 School of Medicine, University of Electronic Science and Technology of China, No.2006, Xiyuan Avenue, High-Tech Zone (West District), Chengdu City, 611731 Sichuan Province People’s Republic of China; 2 Department of Radiation Oncology, Sichuan Cancer Hospital and Institute, Sichuan Cancer Center, School of Medicine, University of Electronic Science and Technology of China, No.55, South Renmin Avenue Fourth Section, Chengdu City, 610041 Sichuan Province People’s Republic of China; 3 Radiation Oncology Key Laboratory of Sichuan Province, No.55, South Renmin Avenue Fourth Section, Chengdu City, 610041 Sichuan Province People’s Republic of China

**Keywords:** Cervical squamous cell cancer, Nonnegative matrix factorization, Immune checkpoint inhibitors, Subtype, Immunophenotype, Immunotherapy response

## Abstract

**Background:**

The main limitation of current immune checkpoint inhibitors (ICIs) in the treatment of cervical cancer comes from the fact that it benefits only a minority of patients. The study aims to develop a classification system to identify immune subtypes of cervical squamous cell carcinoma (SCC), thereby helping to screen candidates who may respond to ICIs.

**Methods:**

A real-world cervical SCC cohort of 36 samples were analyzed. We used a nonnegative matrix factorization (NMF) algorithm to separate different expression patterns of immune-related genes (IRGs). The immune characteristics, potential immune biomarkers, and somatic mutations were compared. Two independent data sets containing 555 samples were used for validation.

**Results:**

Two subtypes with different immunophenotypes were identified. Patients in sub1 showed favorable progression-free survival (PFS) and overall survival (OS) in the training and validation cohorts. The sub1 was remarkably related to increased immune cell abundance, more enriched immune activation pathways, and higher somatic mutation burden. Also, the sub1 group was more sensitive to ICIs, while patients in the sub2 group were more likely to fail to respond to ICIs but exhibited GPCR pathway activity. Finally, an 83-gene classifier was constructed for cervical SCC classification.

**Conclusion:**

This study establishes a new classification to further understand the immunological diversity of cervical SCC, to assist in the selection of candidates for immunotherapy.

**Supplementary Information:**

The online version contains supplementary material available at 10.1186/s12967-021-02894-3.

## Background

Cervical cancer ranks as the fourth most common female malignancy and the fourth leading cause of cancer mortality in women worldwide [1, 2], while low- and middle-income countries account for 90% of the deaths [3, 4]. Although the application of screening and human papillomavirus (HPV) vaccination provide effective prevention for cervical cancer, the imbalance of regional development leads to cervical cancer which will still be a serious health problem in the coming decades [5]. For patients with stage I–III, 15–61% of women will still experience metastatic disease within the first 2 years after completing treatment [6]. Once the disease progresses, second-line and later treatment options are limited, and patients often have a poor prognosis [7].

In recent years, the promising responses of immunotherapy based on inhibitors targeting cytotoxic T lymphocyte antigen 4 (CTLA-4), programmed death receptor 1 (PD-1), or its ligand (PD-L1) have brought revolutionary changes to the treatment of a variety of cancers. It has now become an important issue for cervical cancer. In June 2018, the Food and Drug Administration has approved pembrolizumab for the treatment of recurrent or metastatic cervical cancer based on the preliminary results of the Phase II study of KEYNOTE-158 [8]. To date, the objective response rate (ORR) of immune checkpoint inhibitors (ICIs) for cervical cancer varies from 4% to 26.3% [7, 9–11], with over 80% of responding patients obtaining long-lasting response (> 6 months) [7]. The major limitation of immunotherapy comes from the fact that it benefits only a minority of patients. In consideration of the economic burden and toxicity of ICIs, it is important to identify suitable patients who benefit from ICIs and combination therapy. Still, little is known about how to use the immune-related features of cervical cancer to tailor appropriate immunotherapy for different patients.

On the one hand, almost all cervical cancers are driven by the infection of HPV [12] and are therefore considered to be naturally immunogenic. Meanwhile, HPV mediates a variety of mechanisms to evade innate and adaptive immune responses, making the complex tumor microenvironment (TME) [13]. Based on limited evidence, some scholars believe that the immune status of local TME may play an important role in the relatively low response rate of HPV-related tumors [14, 15]. Squamous cell carcinoma (SCC) is the most common histological subtype accounting for 75% of all cervical cancers [16]. We speculate that the cervical SCC can be further divided into subtypes with distinct immune states according to molecular patterns, which may provide evidence for individualized patient immunotherapy. As one of the source separation techniques, non-negative matrix factorization (NMF) can help separate the molecular characteristics of tissue partitions from the measurement data of tumor samples. It is particularly suitable for biological data because it restricts all sources to be positive in nature, thereby identifying the paradigm of positive gene expression, rather than pairwise differences between tissue types [17]. In this study, a real-world cervical SCC cohort was classified by the NMF based on expression profiles of immune-related genes (IRGs). Subsequently, two subtypes with different prognoses and immunophenotypes were identified and then validated in two public cohorts. The various biological characteristics and the sensitivity to ICIs of each subtype were also described. Finally, an 83-gene-based classifier was constructed to determine the cervical SCC classification.

## Methods and materials

### Real-world patients and samples sequencing

A total of 36 cervical SCC patients who underwent concurrent radiochemotherapy in Sichuan Cancer Hospital (SCCH) between 2013 and 2018 were included in the training cohort according to the enrollment criteria described in our previous study [18]. The protocol was approved by the ethics committee of Sichuan Cancer Hospital and carried out according to the principles of the Declaration of Helsinki. Informed consent was obtained from all patients in the training cohort for the acquisition and use of tissue samples and clinical data.

The 36 formalin-fixed and paraffin-embedded (FFPE) samples were obtained from patients in the training set. The RNA sequencing (RNA-seq) process was conducted as described previously [18]. In this study, we further performed whole-exome sequencing (WES) on the same batch of samples. For each tumor FFPE sample, genomic DNA (gDNA) was extracted with the GeneRead DNA FFPE Kit (QIAgen, Germany) according to the manufacturer’s protocol. The purity and concentration of gDNA were measured using a NanoDrop 2000 Spectrophotometer (Thermo Scientific, USA). High-quality gDNA was sheared with an M220 ultrasonicator (Covaris, USA). Library preparations were performed with KAPA Hyper Prep Kit (KAPA Biosystems, USA). Enriched exome libraries were sequenced on the Illumina NovaSeq 6000 platform. The paired-end reads from the raw FASTQ file were aligned to the hg19 reference genome using BWA-MEM (default parameters, v0.7.15) [19] to generate a binary sequence alignment map (BAM) file, and the duplicate reads were marked and removed using Picard tools (https://broadinstitute.github.io/picard/). Single nucleotide variations (SNVs) and insertions and deletions (INDELs) were called using VarDict [20]. ANNOVAR [21] was used to annotate the variants for further analysis.

### Collection of public data and processing

This study included two independent validation cohorts with clinical information, one of which is the RNA-seq dataset (raw counts) of 255 patients with cervical SCC in the Cancer Genome Atlas (TCGA) Cervical Squamous Cell Carcinoma and Endocervical Adenocarcinoma (CESC) project, retrieved from the Genomic Data Commons (GDC) Legacy Archive (https://portal.gdc.cancer.gov/legacy-archive). The data selection criteria and processing procedures have been described in a previous study [18]. In this study, we further downloaded the somatic mutation data (MAF file) of the aforementioned patient cohort.

As another validation set of this study, the dataset GSE44001 [22] was collected from Gene Expression Omnibus (GEO, http://www.ncbi.nlm.nih.gov/geo/). This mRNA microarray dataset was based on the Platform GPL14951 (Illumina HumanHT-12 WG-DASL V4.0 R2 expression beadchip) and included 300 patients with early cervical cancer (FIGO stage I–II). The raw data (CEL files) from GSE44001 were normalized using the quantile normalization method in the “limma” package, annotated with the “illuminaHumanv4” package before further analysis.

### Selection of immune-related genes associated with prognosis

A comprehensive list of IRGs was obtained from the Immunology Database and Analysis Portal (ImmPort) database (https://www.immport.org), which contained genes closely related to the immune process [23]. Also, we added immune cell-specific signatures according to previous studies [24–28]. Then the univariate Cox proportional hazards model was used to screen out IRGs that are statistically significantly related to the overall survival (OS) of patients in the training set, and finally, genes with p-value < 0.05 were selected as candidates for the NMF analysis.

### Identification and validation of cervical SCC subtypes by NMF

Before performing NMF, candidate IRGs with lower absolute median difference (MAD) values (≤ 0.5) in the corresponding patient cohort were excluded. Mathematically, the gene expression matrix A is considered to be essentially high-dimensional data with the expression levels of N genes in M samples. By applying the NMF algorithm, the matrix was factorized into 2 nonnegative matrices W and H (i.e., A ≈ W × H), where the sizes of matrix W and matrix H are N × k and k × M, respectively [29]. Matrix A was continuously iteratively decomposed, and its outputs were finally integrated into a consensus clustering of samples with k classifications. In this study, the “NMF” package [30] was used to cluster the SCCH cohort for ranks 2 to 6 and the ‘brunet’ algorithm was employed with 200 iterations. The cophenetic correlation coefficients and silhouette scores were directly obtained to determine the optimal rank. After determining number 2 as the best rank, the NMF was run again with rank 2 and performed 200 iterations. To verify the reliability of NMF classification, the same algorithm was also applied to TCGA and GEO cohort by using the same candidate IRGs.

### Comparison of immune-related characteristics between different subtypes

The stromal and immune scores, which respectively represent the proportion of stromal cells and immune cells in tumor samples, were calculated using the ESTIMATE R package. ESTIMATE score is the sum of stromal and immune scores, which is used to infer tumor purity in tumor tissue [31]. Besides, another method used in this study to predict immune infiltration was single-sample Gene Set Enrichment Analysis (ssGSEA), which computed a normalized enrichment score (NES) to quantify the relative abundance of each immune cell type in the tumor microenvironment [32, 33]. A total of 28 immune cell types and corresponding gene signatures were obtained from an online database, The Cancer Immunome Atlas (TCIA, https://tcia.at/) [24]. Besides, we compared the performance of the following biomarkers between different subtypes: immune infiltration score (IIS), T cell infiltration score (TIS), cytolytic activity (CYT), antigen processing and presenting machinery (APM) score, tumor mutational burden (TMB), immune-checkpoint signatures, interferon gamma (INFG) signature and CD8. The IIS for a sample was defined as the mean of the NES of the adaptive immune cells and innate immune cells, while the TIS was a mean score of the following T cell types: activated CD8 + T, T helper, effector memory T cell, central memory T cell, Th1, Th2, Th17, and Treg cells [26]. The CYT was measured as the geometric mean of expression values of granzyme A (GZMA) and perforin (PRF1), which are significantly up-regulated with CD8 + T cell activation [34]. It has been demonstrated that antigen presentation plays a role in the response to ICIs [35]. Using gene set variation analysis (GSVA) function from the “GSVA” package, the APM score was calculated based on a list of antigen presentation related gene signatures (HLA-A, HLA-B, HLA-C, TAP1, TAP2, TAPBP, ERAP1, ERAP2, CANX, CALR, B2M, PDIA3, PSMB5, PSMB6, PSMB7, PSMB8, PSMB9, and PSMB10) [36]. Then, the efficiency of antigen processing and presenting was assessed using normalized APM scores from 0 to 1. The TMB was calculated according to the number of non-synonymous alterations (single nucleotide variants and indel mutations) per megabase (Mb). The immune-checkpoint signatures used genes CD274 (also known as PD-L1), CTLA4, HAVCR, LA3, PDCD1 (also known as PD-1), and PDCD1LG2 (also known as PD-L2) [37]. The INFG signature including CXCL10, CXCL9, HLA-DRA, IDO1, IFNG, and STAT1 [38]. The CD8 signature used CD8A and CD8B.

### Functional analysis by gene set enrichment analysis (GSEA)

Differential expression genes (DEGs) among different cervical SCC subtypes in the SCCH cohort were identified using the “edgeR” package [39, 40]. The genes with p-value < 0.01, false discovery rate (FDR, also known as Benjamini–Hochberg adjusted p-values) < 0.05, and absolute log2 fold change (logFC) > 1.0 were defined as DEGs. Next, genes were ranked by logFC in descending order and then were computed in the “clusterProfiler” package by the GSEA function [41]. The custom gene sets were downloaded from the Molecular Signature Database (MsigDB) v7.2 [42, 43]. Significantly enriched Gene Ontology (GO) biological pathways and Kyoto Encyclopedia of Genes and Genomes (KEGG) pathways were filtered based on a cutoff of FDR < 0.05. NES was used to rank the significantly enriched gene sets.

### Comparison of somatic variations between different subtypes

To maintain consistency with the TCGA database, the somatic mutation information of the SCCH cohort was converted from ANNOVAR annotated file into the MAF file. This process was implemented using the “annovarToMaf” function in the “maftools” package [44]. The MAF file of the TCGA cohort was obtained from the database, as mentioned earlier. In total, mutational data from WES was available for 32 samples in the SCCH cohort and 237 samples in the TCGA cohort. We then compared whether differences exist in mutation frequencies between different subtypes.

### Construction of classifier and performance validation

The DEGs identified above were used as candidate genes to construct our classifier. According to the results of the univariate Cox proportional hazards model, those DEGs that were statistically significantly related to the OS of the SCCH cohort were selected to construct the classifier. Next, we used the genes selected above as variables to determine the optimal “mtry” and “ntree” parameters, and perform tenfold cross-validation. Finally, a random forest classifier based on 500 trees was constructed using the R package “randomForest” [45]. The performance of our classifier to distinguish cervical SCC subtypes was verified in two validation cohorts, and the specificity and sensitivity of the classifier were calculated via the receiver operator characteristic (ROC) curve by using the “pROC” package [46].

### Prediction of the response of each subtype from immunotherapy

To predict the efficacy of immunotherapy in different subtypes, an unsupervised subclass mapping method SubMap (GenePattern, v.3) [47] was used to evaluate correspondence or commonality by measuring the similarity of gene expression profiles between our subtypes and a group of immunotherapy-treated patients. These available patients were from the metastatic melanoma cohort and treated with anti-CTLA-4 or anti-PD-1 antibodies at the University of Texas (UT) MD Anderson Cancer Center [48]. The Bonferroni adjusted p-value was used to assess the extent of the similarity, the smaller the p-value, the greater the similarity. The results of the SubMap analysis were visualized with the “complexHeatmap” package [49].

### Identification of genetic characteristics of subtypes

To evaluate the distribution characteristics of genes in each subtype of cervical SCC and screen the characteristic genes highly correlated with the subtype, a weighted gene co-expression network analysis (WGCNA) was performed on the expression matrix of DEGs by using the “WGCNA” package [50, 51]. First, eliminate outliers to ensure the reliability of the co-expression network results, and then determine the optimal soft threshold based on the standard scale-free model fitting index R^2^. Based on this, the matrix is converted into a topological overlap matrix, the corresponding dissimilarity was calculated. The module eigengenes were calculated to evaluate the correlation between the module and the subtype, and finally, the hub genes from the module most closely related to the subtype were extracted.

### Statistical analysis

All statistical analyses and graphics were performed by using R software (R version 4.0.3). The associations of clinical characteristics between the training set and validation sets were examined by the chi-square test or Fisher’s exact test. The distributions of immune-related characteristics between groups were estimated and tested by the Wilcoxon rank-sum test. Principal component analysis (PCA) was performed using the “FactoMineR” [52] and “factoextra” [53] packages. The “pheatmap” [54] and “complexHeatmap” [49] packages were used for heat maps. Kaplan–Meier curves and log-rank tests were employed to analyze progression-free survival (PFS) and OS rates in the “survival” package. The Cox proportional hazards regression model has also performed in the package “survival” [55]. The package “forestplot” was used for the presentation of the results of the univariable and multivariable analysis [56]. The correlative relationships between immune-related scores were evaluated using Pearson correlation via the package “ggstatsplot” [57]. These scores with a p-value < 0.05 and Pearson correlation coefficient > 0.5 were considered to be strongly correlated. All statistical tests were two-sided.

## Results

### Cohort characteristics

**Fig. 1:**
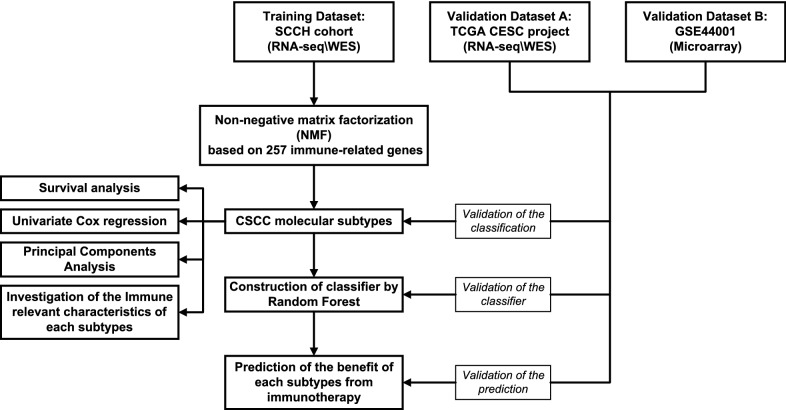
Flowchart of the study design

The study design and workflow are indicated in Fig. 1. Totally 591 patients in three independent cohorts were included in this study. The SCCH and TCGA cohort included both RNA-seq data (n = 36 and 255, respectively) and somatic mutation data (n = 32 and 237, respectively) with full clinical follow-up data, while the GEO cohort contained microarray transcriptomics data from 300 patients with PFS status available only. The baseline characteristics of these patients were summarized in Additional file 2: Table S1. In the SCCH cohort, there was no FIGO stage I patient, and all patients received concurrent chemoradiotherapy, while 49% of the patients in the TCGA cohort were FIGO stage I and the main treatment was a hysterectomy. All patients in the GEO cohort were FIGO I-II stage, and 58.3% of the patients underwent surgery alone.

### Immune-related subtypes in SCCH, TCGA, and GEO cohorts

**Fig. 2:**
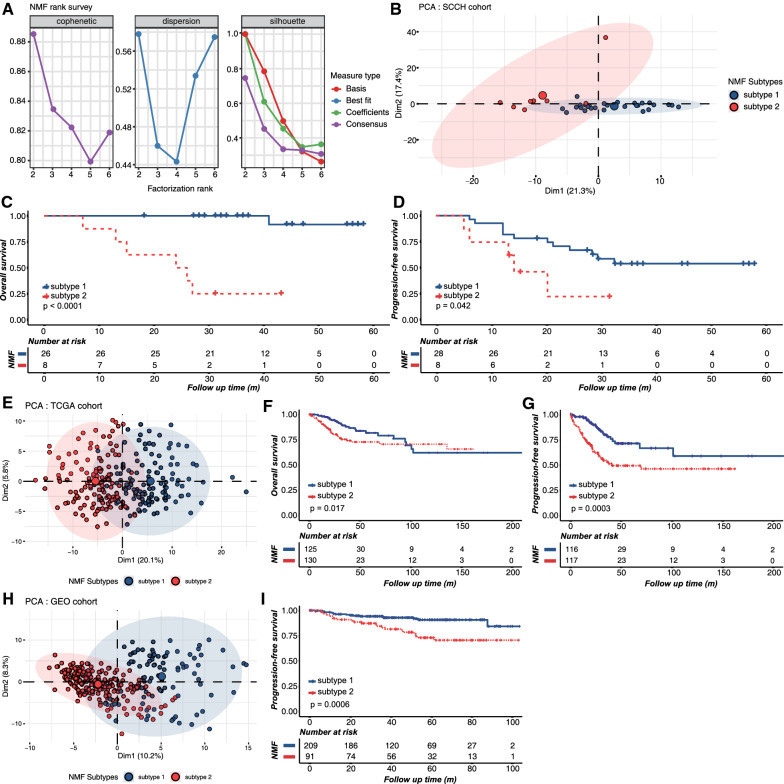
Identification of cervical SCC subtypes with distinct outcomes using NMF consensus clustering. **A** Estimation of the factorization rank (2 to 6; x-axis) using cophenetic, dispersion, and silhouette coefficients (y-axis). There is a large decrease in the stability between rank 2 and 3, indicating that rank 2 has the optimal robustness. **B** PCA analysis of two subtypes from the SCCH cohort. Kaplan–Meier curves for **C** overall survival (OS) and **D** progression-free survival (PFS) show the distinct outcome between subtype 1 and subtype 2 in the validation set. **E** PCA plots of two subtypes in the TCGA cohort. Kaplan–Meier curves for **F** OS and **G** PFS show the distinct outcome between subtype 1 and subtype 2 in the TCGA cohort. **H** PCA plots of two subtypes in the GEO cohort. **I** Survival analysis of the different subtypes in the GEO cohort. The statistical significance of prognosis was determined by a log-rank test

Combining ImmPort and immune cell-specific signatures, a total of 2885 IRGs were obtained (Additional file 2: Table S2), of which 257 genes were significantly correlated with OS in the SCCH cohort (p < 0.05). Using the expression profiles of these 257 IRGs, we performed NMF to cluster the training set. The optimal number of clusters was two, which was decided by cophenetic correlation coefficients, dispersion coefficients, and mean value of silhouette width, as shown in Fig. 2A and Additional file 1: Figure S1 (two subtypes were designated as sub1 and sub2). PCA confirmed that there were robust differences between the gene expression profile of two subtypes (Fig. 2B), and Kaplan–Meier analysis revealed that patients in sub1 had significantly longer OS (p < 0.0001; Fig. 2C) and PFS (p = 0.042; Fig. 2D). To verify the reproducibility of the findings in the SCCH cohort, the above 257 IRGs were used to perform NMF clustering in two independent validation cohorts. As well, the TCGA and GEO cohorts were divided into two subtypes (Additional file 1: Figures S2, S3). The PCA analysis also confirmed the difference in gene expression profile among the two subtypes (Fig. 2E, H). Consistently, survival analysis showed that the prognostic difference between the two subtypes was statistically significant (p < 0.05; Fig. 2F, G, I). Additionally, univariate Cox proportional hazards analysis demonstrated that the acquired new classification was significantly related to prognosis in all three cohorts (sub2 vs. sub1; SCCH cohort for OS: hazard ratio [HR] = 25.64, 95% CI 4.18–53.17, p = 0.001; TCGA cohort for OS: [HR] = 1.84, 95% CI 1.11–3.07, p = 0.019; GEO cohort for PFS: [HR] = 2.92, 95% CI 1.54–5.53, p = 0.001). Multivariate analysis was then used to adjust for potential confounding factors in the baseline characteristics of all three cohorts (e.g., tumor diameter, FIGO stage, and TNM stage), and found that the new classification remained an independent prognostic predictor (Additional file 1: Figure S4). Accordingly, all three cohorts were consistently classified into two subtypes with significantly different prognostic risks. The distribution of clinicopathological characteristics and the different expression patterns of 257 metagenes in the two subtypes are shown in Additional file 1: Figures S5, S6, and S7.

### Differences of immune-related characteristics between cervical SCC subtypes

**Fig. 3:**
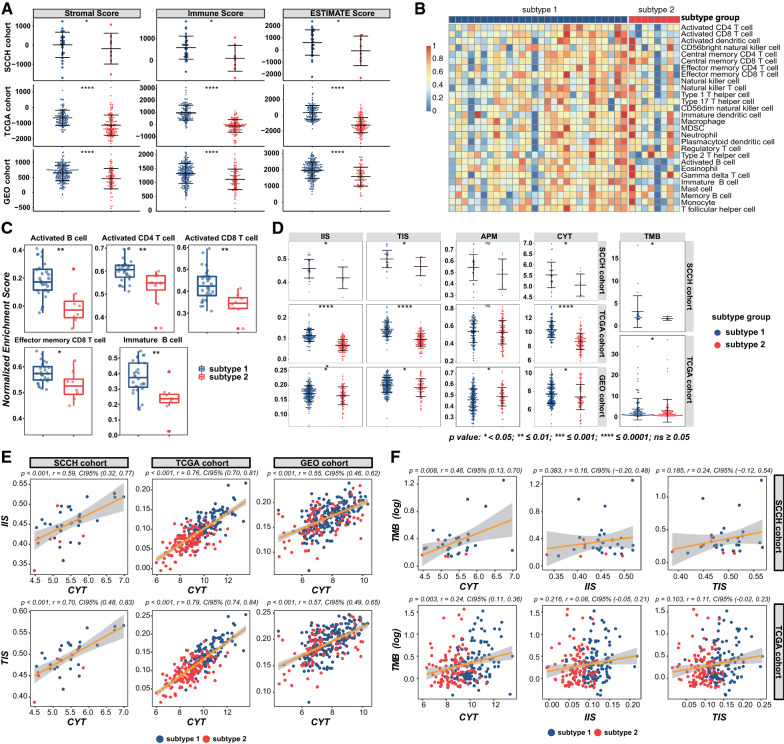
Immune characteristics of two subtypes in the training set and validation set. **A** Comparisons of the stromal scores, immune scores, and ESTIMATE scores for the two subtypes in all three cohorts. **B** Heatmap showing the abundance of immune cells for different subtypes in the SCCH cohort. The relative abundance of each cell type was standardized using zero-mean normalization. **C** The boxplot representation of immune cells with significant differences in distribution between the subtype 1 and subtype 2 in the SCCH cohort. **D** Evaluation of the level of 4 important immune scores and tumor mutational burden (TMB) between two subtypes in the training set and validation set. The statistical difference in **A**, **C**, **D** was compared through the Wilcoxon rank-sum test. (ns represents no significance, * p-value < 0.05, **p ≤ 0.01, *** p-value ≤ 0.001, **** p ≤ 0.0001). **E** Correlation between cytolytic activity (CYT), immune infiltration score (IIS), and T cell infiltration score (TIS) in three cohorts. **F** Correlation between cytolytic activity (CYT), immune infiltration score (IIS), T cell infiltration score (TIS), and TMB in the training set and validation set. The p-value and Pearson’s correlation coefficients with a 95% confidence interval shown at the top of each plot

To explore the heterogeneity of immune characteristics between the two subtypes, a series of immune-related algorithms were applied for analysis. Using ESTIMATE, we found that the stromal, immune, and ESTIMATE scores of sub1 in the SCCH cohort were significantly higher than sub2 (p < 0.05; Fig. 3A, upper panel). Next, the verification results in the TCGA and GEO cohorts were also consistent, that is, the stromal, immune, and ESTIMATE scores for sub1 were considerably higher than those for sub2 (p < 0.0001; Fig. 3A, middle and lower panels). These results suggesting that the TME in sub1 contained a higher number of immune components in both the training and validation set. To further clarify the inherent microenvironment differences of different subtypes, the ssGSEA was applied to measure the abundance of 28 immune cell types, TIS, IIS, APM score, CYT, and TMB were also calculated accordingly. The heatmap was generated to visualize the relative infiltration of immune cell populations across three cohorts (Fig. 3B and Additional file 1: Figure S8A). The abundance of activated CD4 + T cells and effector memory CD8 + T cells in sub1 was significantly higher than that of sub2 in all three cohorts. In the SCCH and TCGA cohort, activated CD8 + T cell, activated B cell, and immature B cell also showed a consistent distribution, that is, the distribution in sub1 was significantly higher than that in sub2. The remaining cells were still showing a higher tendency in sub1, except for mast cell and γδ T cell. This finding was then confirmed in the TCGA cohort (Fig. 3C and Figure S8B). IIS and TIS represent the overall infiltration degree of immune cells and T cells respectively. Although the distribution pattern of infiltrating cells in the GEO cohort between the two subtypes was not exactly similar to that in the SCCH and TCGA cohorts, the sub1 in all three cohorts exhibited higher IIS and TIs, suggesting that the overall level of immune infiltrating cells was consistent among the training and validation cohorts (Fig. 3D, left panel). We also observed a higher level of CYT and an increased TMB in the sub1, while the APM score did not show a similar trend in the three cohorts (Fig. 3D, middle and right panels). The significantly different immune scores were selected for the next correlation analysis. As shown in Fig. 3E, F, Pearson’s correlation analysis was used to investigate the relationships between these significantly different immunity scores in the training and validation set. In all three cohorts, CYT has a strong positive correlation with IIS and TIS (p < 0.001, r = 0.55–0.79; Fig. 3E). In the SCCH cohort, TMB and CYT were moderately positively correlated (p = 0.008, r = 0.46; Fig. 3F, upper left panel), but there was no correlation with IIS or TIS (p > 0.05; Fig. 3F, upper middle and right panel). In the TCGA cohort, TMB was weakly positively correlated with CYT (p = 0.003, r = 0.24; Fig. 3F, lower left panel), but there was still no correlation with TIS and IIS (p > 0.05; Fig. 3F, lower middle and right panel).

**Fig. 4:**
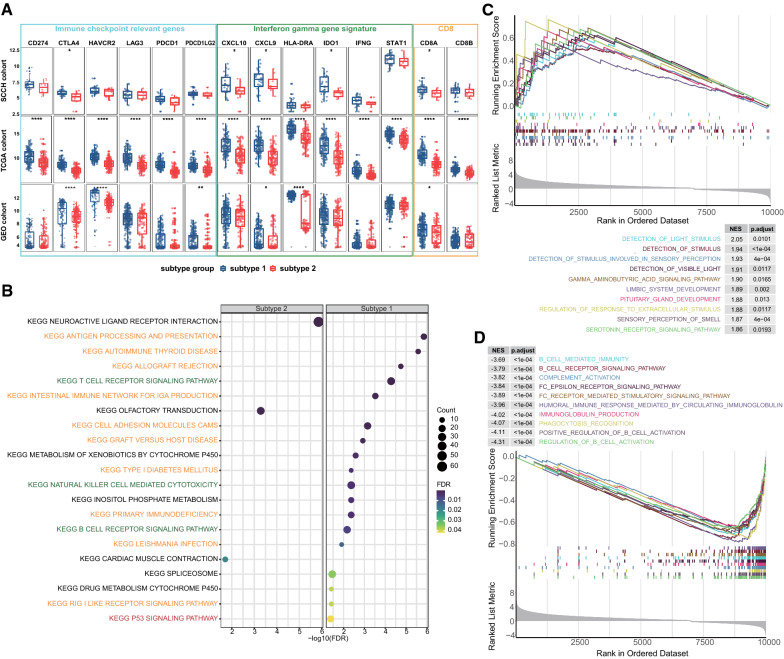
The expression level of selected transcriptomic signatures and pathways enriched in two subtypes. **A** Expression level (normalized count) of transcriptomic signatures among two subtypes in SCCH, TCGA and GEO cohorts (Wilcoxon rank-sum test, *p-value < 0.05, **p ≤ 0.01, ***p-value ≤ 0.001, ****p ≤ 0.0001). **B** The KEGG enrichment pathways ranked by false discovery rate (FDR). Orange represented immune response-associated pathways, green represented immune cell-associated pathways, and red represented tumor suppressor pathways. The top 10 enrichment pathways ranked by normalized enrichment score (NES) in **C** subtype 1 and **D** subtype 2

The gene-expression-profiling-based biomarkers among different subtypes were also evaluated. In the sub1 group of all the three cohorts, the expression levels of CTLA4, CXCL9, IDO1, and CD8A were significantly higher. The expression level of all biomarker genes in sub1 of the TCGA cohort was significantly higher than that of sub2. In the SCCH and GEO groups, a rising trend of the remaining genes including CD274 and PDCD1 in sub1 were observed, although the difference was not statistically significant (Fig. 4A). Therefore, we defined the sub1 with a high degree of cytotoxic T cell infiltration and increased cytolytic activity as immune-enriched subtype, and sub2 with low immune infiltrates as immune-desert subtype.

### Differences of biological functions between cervical SCC subtypes

We next sought to investigate the biological changes associated with each subtype using the GSEA. A total of 21 KEGG pathways and 138 GO biological pathways met our strict threshold (FDR < 0.05). We listed all the significantly enriched KEGG pathways between sub1 and sub2 in ascending order of FDR from top to bottom (Fig. 4B). Additional file 2: Table S3 listed the detailed information of the KEGG pathways. It can be seen that the pathways enriched in sub1 include innate immunity, antigen processing and presentation, cellular immunity, humoral immunity, and autoimmune diseases. The enrichment pathways of sub2 were mainly related to signal transmembrane conduction. Similarly, among the 92 GO pathways enriched in the sub1, most of them were immune regulatory pathways related to cellular or humoral immunity (Additional file 2: Table S4). As shown in Fig. 4C, the top 10 pathways included positive regulation of B cell activation, phagocytosis recognition, immunoglobulin production, Fc receptor-mediated stimulatory signaling pathway, complement activation, etc. The 46 GO pathways enriched in the sub2 were mainly related to the G protein-coupled receptor (GPCR) signaling pathway, GPCR coupled second messenger signaling pathway, and the transmembrane signal transduction mediated by GPCR (Additional file 2: Table S4). As shown in Fig. 4D, the top 10 pathways in sub2 were closely related to the GPCR pathway, including the visual system, olfactory system, stimulus perception of the sensory system, and neuroendocrine system.

Collectively, these findings indicate that the sub1 was characterized by significantly enriched immune-related signaling pathways, involving immune cell signals, immune response signals, and interferon-gamma-related signals, etc. The sub2 was characterized by a significantly enriched GPCR signaling pathway and transmembrane signal transduction mediated by GPCR.

### Mutation patterns in different subtypes

**Fig. 5:**
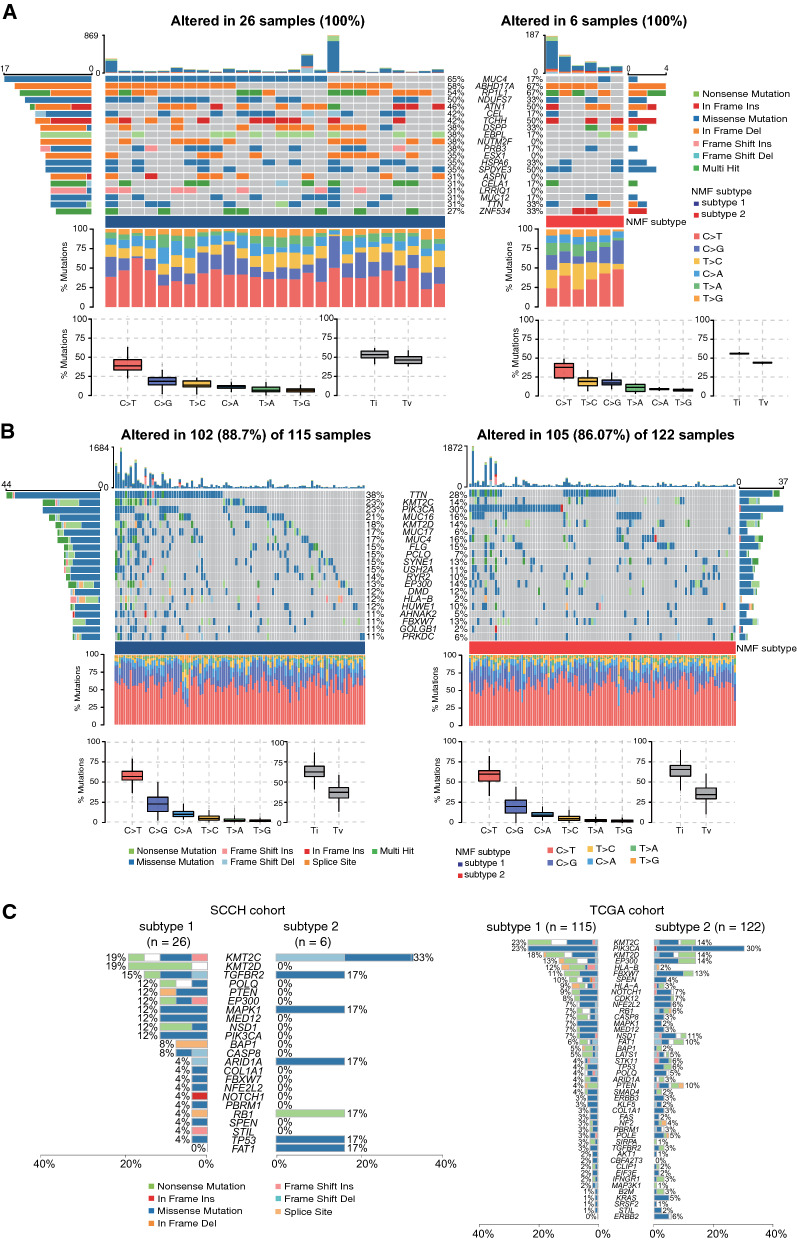
Mutation profiles of the **A** SCCH cohort and **B** TCGA cohort stratified by cervical SCC subtypes. **C** The distribution of driver gene mutation frequencies in subtype 1 and subtype 2 among SCCH and TCGA cohorts, respectively

The somatic mutation distribution of each cohort was investigated and the difference of mutation patterns among cervical SCC subtypes was compared. In the SCCH cohort, the top 20 mutation genes in sub1 were shown in the upper left panel of Fig. 5A, while mutation proportions of the same genes were re-ranked in sub2 and illustrated in the upper right panel of Fig. 5A. Missense mutation accounted for the most fractions in both subtypes. The most frequent genes were MUC4 (65%), ABHD17A (58%), RP1L1 (54%) and NDUFS7 (50%) in sub1, while the corresponding proportion in sub2 was 17, 67, 67, and 33%, respectively. SNVs analysis showed that transition mutations, specifically C to T, were prominent in both subtypes; C to G ranks second in sub1 and third in sub2 (Fig. 5A, lower left and right panel). Similarly, the most frequent mutation categories in the TCGA cohort were also a missense mutation, and C to T was the most common transition mutation among subtypes (Fig. 5B). Also, by evaluating the mutation frequency distribution of 45 cervical SCC driver genes in different subtypes [58], we found that the immune-enriched subtype was associated with a high mutation frequency of multiple driver genes (e.g., KMT2D, PIK3CA, PTEN, HLA-B), while TP53, ARID1A, FAT1, and ERBB2 showed a higher mutation frequency in the immune-desert subtype (Chi-square test; Additional file 2: Table S5). Figure 5C showed the distribution of driver genes in cervical SCC subtypes among the SCCH cohort and the TCGA cohort. The enriched P53 signaling pathway in the sub1 group and the higher mutation frequency of TP53 in the sub2 group also corresponded to the different prognosis between the two groups.

### Classifier construction and validation

**Fig. 6:**
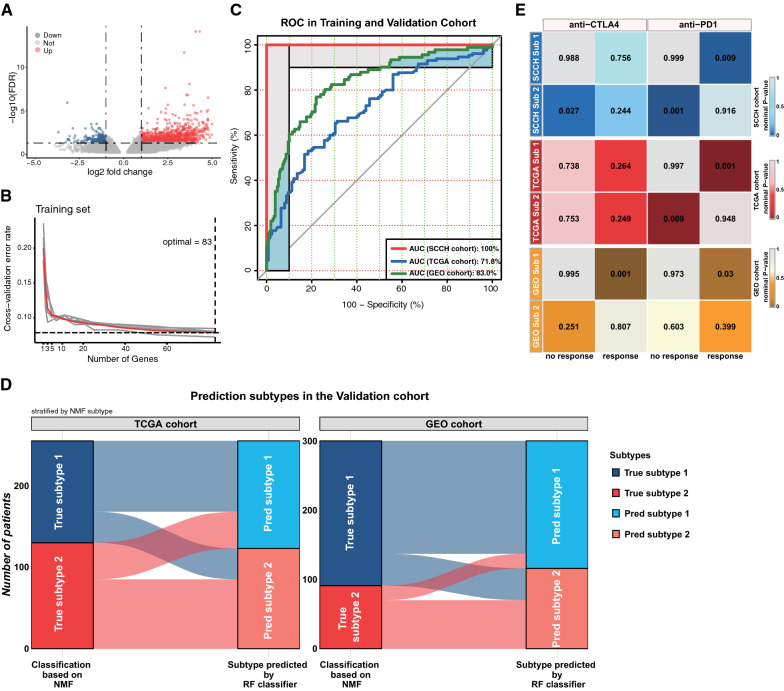
Construction of classifier and prediction of immunotherapeutic response. **A** Volcano plots of differentially expressed genes (DEGs) between subtype 1 and subtype 2 in the SCCH cohorts. Genes with false discovery rate (FDR) < 0.05 and |log2 fold change|> 1 are considered as DEGs. The horizontal dashed line indicates the FDR of 0.05. The vertical dashed lines indicate the log2 fold change values of -1 and 1. Blue points: down-regulated genes; Gray points: non-differential genes; Red points: up-regulated genes. **B** Error rate curve based on tenfold cross-validation using an increased number of variables. The vertical dashed lines indicate that the lowest error rate was obtained when the number of variables is 83, which is the best model. **C** ROC curves in the SCCH cohort (red line), TCGA cohort (blue line), and GEO cohort (green line). The area under the curves (AUC) was used to evaluate the accuracy of the classifier. **D** Concordance of cervical SCC subtypes prediction between the 83-gene-based classifier and original classification based on NMF. **E** Subtype 1 shares a high similarity with the PD-1 response group in all three cohorts. Subtype 2 shares a high similarity with the PD-1 no-response group in the SCCH cohort and TCGA cohort. The colors labeled in each cell indicate the Bonferroni adjusted p-values of each subclass association

Between the two subtypes in the training cohort, a total of 1014 DEGs were identified (FDR < 0.05, |log2 FC|> 1.0; Fig. 6A). Using the univariate Cox proportional hazards model, 83 mRNAs significantly related to OS were selected (Additional file 2: Table S6). Based on these genes, the optimal “mtry” parameter (n = 2) and the number of decision trees (n = 500) were first determined. Next, tenfold cross-validation was performed to avoid overfitting and determine the number of variables for the optimal classifier. After repeating the cross-validation 10 times, the classifier with the minimum error was obtained (Fig. 6B). Subsequently, the ROC curve analysis was performed and the area under the ROC curve (AUC) was 100% in the SCCH cohort. The AUC of the TCGA and GEO cohort reached 71.8% and 83%, respectively (Fig. 6C). So far, we have successfully constructed the 83-gene classifier, and have good performance in two independent validation cohorts (Fig. 6D).

### Distinct sensitivity to immunotherapy for cervical SCC subtypes

The heterogeneous immune infiltration patterns and different TMB levels among cervical SCC subtypes suggested that the potential immunotherapy benefits needed to be further explored. A SubMap analysis was then conducted to compare the expression profiles of two cervical SCC subtypes with a published metastatic melanoma cohort containing 56 patients that received anti-PD-1 or anti-CTLA-4 treatment. The result showed that sub1 in the SCCH cohort was highly correlated with the PD-1 response group (Bonferroni correction p-value = 0.009; Fig. 6E, upper right panel), indicating that patients within the sub1 group were more promising to respond to anti-PD-1 therapy. On the contrary, sub2 was significantly correlated with both CTLA-4 and PD-1 no-response groups (Bonferroni correction p-value = 0.027 and 0.001; Fig. 6E, upper panel), suggesting that the sub2 group might be resistant to ICIs. In the TCGA cohort, when comparing cervical SCC subtypes with the immunotherapy groups, a significant relationship was observed between sub1 and PD-1 response groups, and the same between sub2 and PD-1 no-response group (Fig. 6E, middle panel). It showed that patients in the sub1 group were more sensitive to anti-PD-1 therapy, while patients in the sub2 group were more likely to fail to respond to anti-PD-1 therapy. Likewise, the expression profile of sub1 in the GEO cohort had high similarity with both anti-CTLA-4 and anti-PD-1 sensitive groups (Fig. 6E, lower panel).

### Cervical SCC subtypes related genes

To further identify the gene signature of the cervical SCC subtype, a total of 1014 DEGs among different subtypes were evaluated by WGCNA. After removing one sample as an outlier through hierarchical clustering analysis, the rest were used for the construction of the co-expression network (Additional file 1: Figure S9A). By the selected power of β = 4 as the soft-thresholding (Additional file 1: Figure S9B), a total of 8 modules were identified (Additional file 1: Figure S9C). The turquoise module was found to have the highest positive correlation with sub1 (correlation coefficient = 0.74, p < 0.001), and the highest negative correlation with sub 2 (correlation coefficient = − 0.74, p < 0.001). Next, gene significance was calculated to quantify the correlation between individual genes and the subtype, and module membership was calculated to quantify the correlation between the turquoise module and the DEGs expression profile. As shown in Additional file 1: Figure S9D, the module membership was significantly positively correlated with gene significance in subtypes (correlation coefficient = 0.7, p < 0.001). Using the same algorithm to identify the gene modules with the highest subtype correlation in the TCGA and GEO cohorts, 78 and 40 genes were obtained, respectively. A total of 23 overlapping DEGs in the three cohorts were found (Additional file 1: Figure S10A and 10B). Further comparisons showed that the distribution patterns of all 23 genes between the sub1 and sub2 were the same. Among them, CA9, TCHHL1, MGAT5B, and BIRC5 are significantly overexpressed in sub2, while the remaining genes were highly expressed in sub1 (Additional file 1: Figure S11, Additional file 2: Table S7).

## Discussion

Although the current results of ICIs treatment for cervical cancer are encouraging, there are improvements to be made. Identifying suitable patients is expected to further increase the proportion of patients who benefit from ICIs. In this study, we constructed a classification of cervical SCC patients based on 2885 IRGs obtained from relevant publications and public databases. Two subtypes (sub1 and sub2) with distinct prognoses were identified using the NMF method. The sub1 patients showed significantly enriched features about immune cells (CD8/CD4 T cells, B cells) and enhanced cytolytic activity. We observed higher levels of the immune checkpoint, INFG, and CD8 signatures. Similarly, GSEA has identified a series of immune-related signaling pathways up-regulated in the immune-enriched subtype. Also, the immune-enriched subtype exhibited increased TMB and was more sensitive to ICIs, which is consistent with its good prognostic phenotype. Conversely, the immune-desert subtype with a worse prognosis exhibited lower levels of TMB and resistance to anti-PD-1. The repeatability of this classification was further verified through two independent verification sets.

Mounting evidence has identified that the TME plays a key role in the occurrence and development of tumors, as well as profoundly affects the therapeutic efficacy and patient prognosis. Based on the characteristics of TME, immune-activated and immune-suppressed subtypes have been identified in pancreatic cancer [17], hepatocellular carcinoma [59], and head and neck squamous cell carcinoma (HNSCC) [60]. For cervical SCC, immune-enriched subtype and immune-desert subtype were found in this study. Within these subtypes, a favorable prognosis was associated with increased infiltration of activated CD8 + T cells, effector memory CD8 + T cells, activated CD4 + T cells, and plasma cells, and vice versa. The association of these cell infiltrations with DFS and/or OS has been widely confirmed in multiple cancers [61, 62]. Among them, CD8 + T cell infiltration was considered to be the most promising signature related to beneficial clinical outcomes in cervical cancer [63].

As expected, CYT, as a measure of CD8 + T cell activation, was significantly up-regulated when ICIs produced clinical responses [64, 65], which was also proved by the results in our study. APM score is calculated based on genes involved in the APM process [66], which could reflect the formation of major histocompatibility complex (MHC) class I molecules and the efficiency of their recognition and killing by CD8 + T cells and NK cells [36]. Defects in the expression of APM components affect the recognition of tumor antigens [35]. Here, no difference in APM score was observed between the two subtypes, implying the ubiquity of HPV-mediated immune escape mechanisms. The significantly enriched RIG-I-like receptors signaling pathway in the sub1, which is responsible for detecting viral pathogens and activating antiviral immunity, was consistent with the baseline characteristics of more HPV + patients included in the sub1. Besides, a series of immune-related pathways were significantly enriched in the sub1, and perform multiple functions including inducing autoimmune response, cytotoxic activation, various immune cell activation, antigen processing and presentation, host defense, and immune monitoring. There was no immune-related pathway observed in sub2. The enrichment of the GPCR signaling pathway explains the poor prognosis of the sub2, which promotes the angiogenesis, invasion, migration, and metastasis of a variety of malignancies [67]. From these results, we found that the TME of cervical SCC not only shares the commonality as other solid tumors, but also possesses its own features due to the particularity of its etiology [13].

Mutation burden as a biomarker of response to ICIs has received widespread attention in recent years. The prospective clinical evidence involves various solid tumors, such as non-small cell lung cancer [68], small cell lung cancer [69], melanoma [70], bladder cancer [71], glioblastoma [72], colorectal cancer [73]. Our results suggested that the prognosis and the prediction of response to ICIs of patients with high TMB were better than those of patients with low TMB, which is also consistent with the existing clinical results of cervical cancer [74, 75]. This could be due to the more mutations accumulated in the tumor, the more neoantigens are produced [76]. These neoantigens are presented to cytotoxic T cells through the MHC molecules on the surface of tumor cells, resulting in T cell activation and anti-tumor immune response [77]. This explains to a certain extent the weak positive correlation between TMB and CYT. Interestingly, although increased TMB was accompanied by immune-enriched subtype, further analysis showed that there was no correlation between somatic mutations and immune infiltration. Similarly, we have also observed that a higher TMB does not necessarily mean a higher immune infiltration in several tumors, including hepatocellular carcinoma, pancreatic cancer, and HNSCC [59, 60, 78]. It implies the independent predictive value of mutation or neoantigen load.

The newly defined cervical SCC subtypes with distinct immunophenotypes were assessed by the melanoma cohort. We found that patients with the immune-enriched subtype could benefit more from ICIs and may be ideal candidates. For patients with the immune-desert subtype, considering the activity of GPCR and GPCR coupled second messenger signaling pathways in sub2, treatments targeting GPCRs and their coupled downstream signaling molecules may be beneficial to them. Indeed, as more GPCRs with tumors are revealed, treatment targeting GPCRs has become increasingly attractive. Currently, GPCR-targeted agents have been approved for the treatment of advanced prostate cancer and basal cell carcinoma [79]. Our findings suggest that the GPCRs pathway is a potential therapeutic target in cervical SCC. In particular, the possibility of benefiting patients with the immune-desert subtype emphasizes the value of in-depth research on this issue.

Finally, 23 genes closely related to subtypes were identified through WGCNA. Finally, 23 genes closely related to subtypes were identified through WGCNA. Among the overexpressed genes in sub1, 13 genes are known to be closely related to the proliferation and activation of T/B cells and the activation of immune responses (including ICOS, TRAT1, ZAP70, SPN, MS4A1, ITK, CCR7, CD3E), CD28, IL2RB, CCL19, FCRL5, IRF4). UBASH3A is overexpressed in CD8 T cells, and the transcription product negatively regulates T cell signaling [80]. AKR1C1 has been confirmed as a biomarker of cancer-associated fibroblasts in TME [81, 82]. The overexpression of ITM2A, ATP2A3, and tumor suppressor gene SFRP1 is also closely related to a better prognosis of cervical cancer [83–85]. The protein encoded by SPOCK2 is a component of the extracellular matrix, and down-regulation of SPOCK2 indicates a poorer prognosis for prostate cancer [85]. Interestingly, SPOCK2 has not been reported to be associated with cervical cancer, suggesting that SPOCK2 is a valuable research direction. CA9, MGAT5B, TCHHL1, and BIRC5 were found to be significantly overexpressed in sub2. CA9 is considered to be a new specific biomarker for cervical cancer hypoxic cells [86]. Cervical cancer with a high expression of CA9 has a higher rate of local recurrence and distant metastasis [87] and is closely related to the poor prognosis of early cervical cancer [88]. Similarly, MGAT5B is highly correlated with tumor progression and metastasis [89, 90], and microenvironment hypoxia can further stimulate the expression of MGAT5B [91]. Although the relationship between TCHHL1 and cervical cancer remains unclear, the high expression of TCHHL1 plays an important role in promoting the proliferation of squamous cells [92]. Moreover, TCHHL1 was reported to be a target gene of Kruppel-like transcription factor family member KLF4, which is important for activating HPV viral transcription [93], suggesting that TCHHL1 is also a promising potential therapeutic target for cervical SCC. BIRC5 (also known as survivin) has been proven to regulate migration and invasion of a variety of cancer cells, including cervical cancer, and is a well-known target for cancer therapy [94]. More and more studies suggest that the development of BIRC5 specific anti-cancer drugs is making progress [95]. In general, the significantly higher expression of molecules in sub2 in our results explained the worse prognosis of sub2. More importantly, these molecules are promising therapeutic targets for cervical SCC, especially for patients with the immune-desert subtype.

In conclusion, we identified two molecular subtypes of cervical SCC, immune-enriched subtype and immune-desert subtype. This newly constructed 83-gene classification system might aid in predicting the prognosis and immune status of patients, identifying ideal candidates for immunotherapy, and individually specifying treatment strategies. We also provided valuable research directions for those patients who are unlikely to benefit from immunotherapy. The findings of this study warrant further investigation in a larger cohort of cervical SCC undergoing immunotherapy.

## Supplementary Information

**Additional file 1.** **Figure S1.** Heatmap representation of non-negative matrix factorization clustering map from rank2 to 6 in the SCCH cohort. **Figure S2.** (A) The relationship between cophenetic, dispersion, and silhouette coefficientsconcerning 2 to 6 clusters in the TCGA cohort. (B) Non-negative matrix factorization clustering map of rank 2 in theTCGA cohort. **Figure S3.** (A) The relationship between cophenetic, dispersion, and silhouette coefficientsconcerning 2 to 6 clusters in the GEO cohort. (B) Non-negative matrix factorization clustering map of rank 2 in theGEO cohort. **Figure S4.** Forest plot of hazard ratios (HR) for prognostic value assessed by the cervical SCCsubtype classifier and clinicopathological characteristics in the training set and validation set. Error bars represent95% confidence intervals. The vertical grey line represents HR = 1. **Figure S5.** The distribution of clinicopathological characteristics and the different expressionpatterns of 257 metagenes among the two subtypes in the SCCH cohort. **Figure S6.** The distribution of clinicopathological characteristics and the different expressionpatterns of metagenes among the two subtypes in the TCGA cohort. **Figure S7.** The distribution of clinicopathological characteristics and the different expressionpatterns of metagenes among the two subtypes in the GEO cohort. **Figure S8.** The abundance of 28 immune cell types estimated by the ssGSEA algorithm betweensubtype 1 and subtype 2 in the (A) TCGA and (B) GEO cohort. (C) Box plots depicting the distribution of immune celltypes among the two subtypes in all three cohorts. The normalized enrichment score (NES) was compared throughthe Wilcoxon rank-sum test. (* p-value < 0.05, ** P ≤ 0.01, *** p-value ≤ 0.001, **** P ≤ 0.0001). **Figure S9.** The weighted gene co-expression network analysis in the SCCH cohort. (A) Onesample was deleted as an outlier after the hierarchical clustering analysis. (B) The power of β = 4 was selected asthe optimal soft threshold. (C) Identification of the correlation between module eigengenes and subtypes of cervicalSCC. The corresponding correlation and P-value are at the top and bottom of each cell respectively. (D) Correlationsbetween the gene significance and module membership in the turquoise module. **Figure S10.** A total of 23 overlapping DEGs in the three cohorts were found. (A) Venn diagramillustrating the number of overlapping DEGs. (B) Distribution of 23 DEGs in the Volcano plots. **Figure S11.** Box plots depicting the distribution of 23 overlapping DEGs among the two subtypesin all three cohorts. The expression count of DEGs was compared through the Wilcoxon rank-sum test. (* p-value <0.05, ** P≤0.01, *** p-value≤0.001, **** P≤0.0001).

**Additional file 2.** **Table S1.** Baseline clinical features for the cervical SCC patients in the training set and validationset. **Table S2.** The list of immune-related genes (IRGs). **Table S3.** The enriched 21 Kyoto Encyclopedia of Genes and Genomes (KEGG) pathwaysbetween sub1 and sub2. **Table S4.** The enriched 138 Gene Ontology (GO) biological pathways between sub1 and sub2. **Table S5.** The mutation frequencies of 45 driver genes of cervical SCC between sub1 and sub2. **Table S6.** 83 mRNAs significantly related to OS used to construct the random forest classifier. **Table S7.** 23-mRNA signature related to cervical SCC subtypes.

## Data Availability

The raw data used and/or analyzed during the current study are available from the corresponding author on reasonable request.

## Abbreviations

HPV: Human papillomavirus
CTLA-4: Cytotoxic T-lymphocyte-associated protein-4
PD-1: Programmed cell death protein-1
PD-L1: Programmed cell death ligand 1
ORR: Objective response rate
ICIs: Immune checkpoint inhibitors
TME: Tumor microenvironment
SCC: Squamous cell carcinoma
NMF: Nonnegative matrix factorization
IRGs: Immune-related genes
SCCH: Sichuan cancer hospital
FFPE: Formalin-fixed and paraffin-embedded
RNA-seq: RNA sequencing
WES: Whole-exome sequencing
gDNA: Genomic DNA
BAM: Binary sequence alignment map
SNVs: Single nucleotide variations
INDELs: Insertions and deletions
TCGA: The cancer genome atlas
CESC: Cervical squamous cell carcinoma and endocervical adenocarcinoma
GDC: Genomic data commons
GEO: Gene expression omnibus
ImmPort: Immunology database and analysis portal
OS: Overall survival
MAD: Absolute median difference
ssGSEA: Single-sample gene set enrichment analysis
NES: Normalized enrichment score
TCIA: The cancer immunome atlas
IIS: Immune infiltration score
TIS: T cell infiltration score
CYT: Cytolytic activity
APM: Antigen processing and presenting machinery
TMB: Tumor mutational burden
INFG: Interferon gamma
GZMA: Granzyme A
PRF1: Perforin 1
GSVA: Gene set variation analysis
GSEA: Gene set enrichment analysis
DEGs: Differential expression genes
FDR: False discovery rate
logFC: Log2 fold change
MsigDB: Molecular signature database
GO: Gene ontology
KEGG: Kyoto encyclopedia of genes and genomes
ROC: Receiver operator characteristic;
UT: University of Texas
PCA: Principal component analysis
PFS: Progression-free survival
HR: Hazard ratio
CI: Confidence interval
GPCR: G protein-coupled receptor
AUC: Area under the ROC curve
HNSCC: Head and neck squamous cell carcinoma
MHC: Major histocompatibility complex
