# Supplementary material for: Identification of immune subtypes of cervical squamous cell carcinoma predicting prognosis and immunotherapy responses
Source: J Transl Med. 2021 May 24;19:222. doi: 10.1186/s12967-021-02894-3 (PMC8142504; doi:10.1186/s12967-021-02894-3)
Supplement: Supplementary file 1 — Additional file 1. Figure S1. Heatmap representation of non-negative matrix factorization clustering map from rank2 to 6 in the SCCH cohort. Figure S2. (A) The relationship between cophenetic, dispersion, and silhouette coefficientsconcerning 2 to 6 clusters in the TCGA cohort. (B) Non-negative matrix factorization clustering map of rank 2 in theTCGA cohort. Figure S3. (A) The relationship between cophenetic, dispersion, and silhouette coefficientsconcerning 2 to 6 clusters in the GEO cohort. (B) Non-negative matrix factorization clustering map of rank 2 in theGEO cohort. Figure S4. Forest plot of hazard ratios (HR) for prognostic value assessed by the cervical SCCsubtype classifier and clinicopathological characteristics in the training set and validation set. Error bars represent95% confidence intervals. The vertical grey line represents HR = 1. Figure S5. The distribution of clinicopathological characteristics and the different expressionpatterns of 257 metagenes among the two subtypes in the SCCH cohort. Figure S6. The distribution of clinicopathological characteristics and the different expressionpatterns of metagenes among the two subtypes in the TCGA cohort. Figure S7. The distribution of clinicopathological characteristics and the different expressionpatterns of metagenes among the two subtypes in the GEO cohort. Figure S8. The abundance of 28 immune cell types estimated by the ssGSEA algorithm betweensubtype 1 and subtype 2 in the (A) TCGA and (B) GEO cohort. (C) Box plots depicting the distribution of immune celltypes among the two subtypes in all three cohorts. The normalized enrichment score (NES) was compared throughthe Wilcoxon rank-sum test. (* p-value < 0.05, ** P ≤ 0.01, *** p-value ≤ 0.001, **** P ≤ 0.0001). Figure S9. The weighted gene co-expression network analysis in the SCCH cohort. (A) Onesample was deleted as an outlier after the hierarchical clustering analysis. (B) The power of β = 4 was selected asthe optimal soft threshold. (C) Identifica [file 12967_2021_2894_MOESM1_ESM.docx]

**Figure S1：**


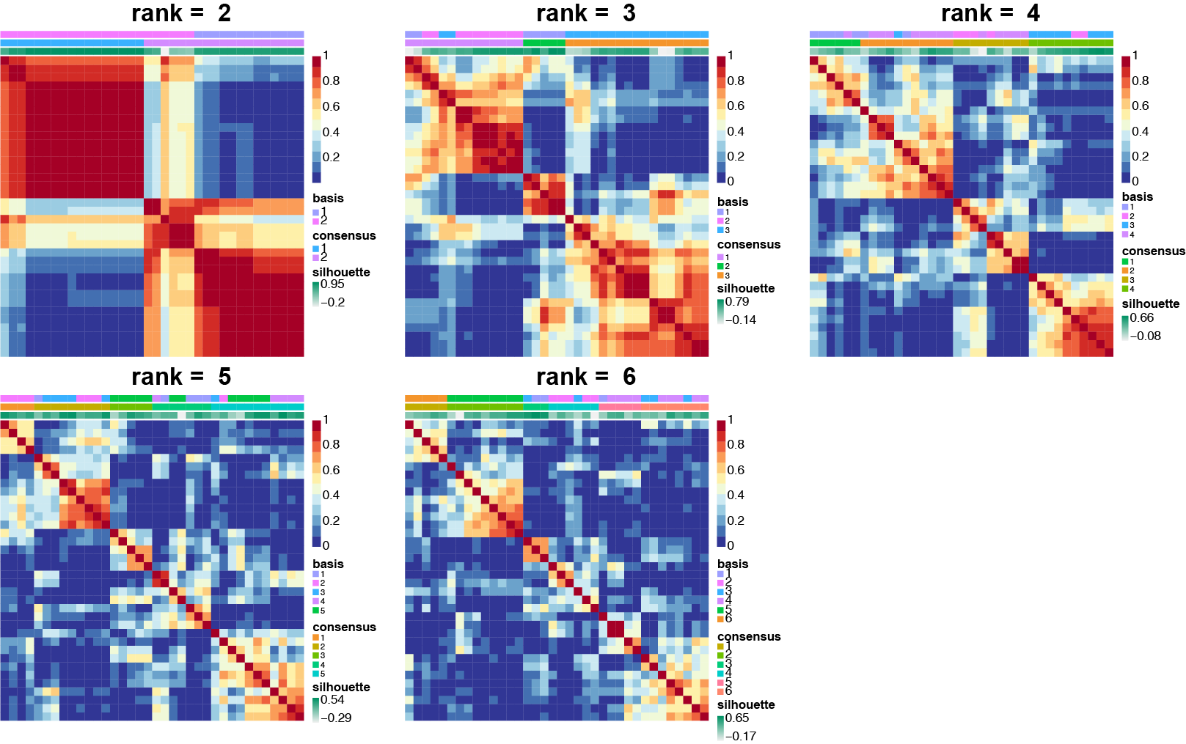


**Figure S1** Heatmap representation of non-negative matrix factorization clustering map from rank 2 to 6 in SCCH cohort.

**Figure S2**：


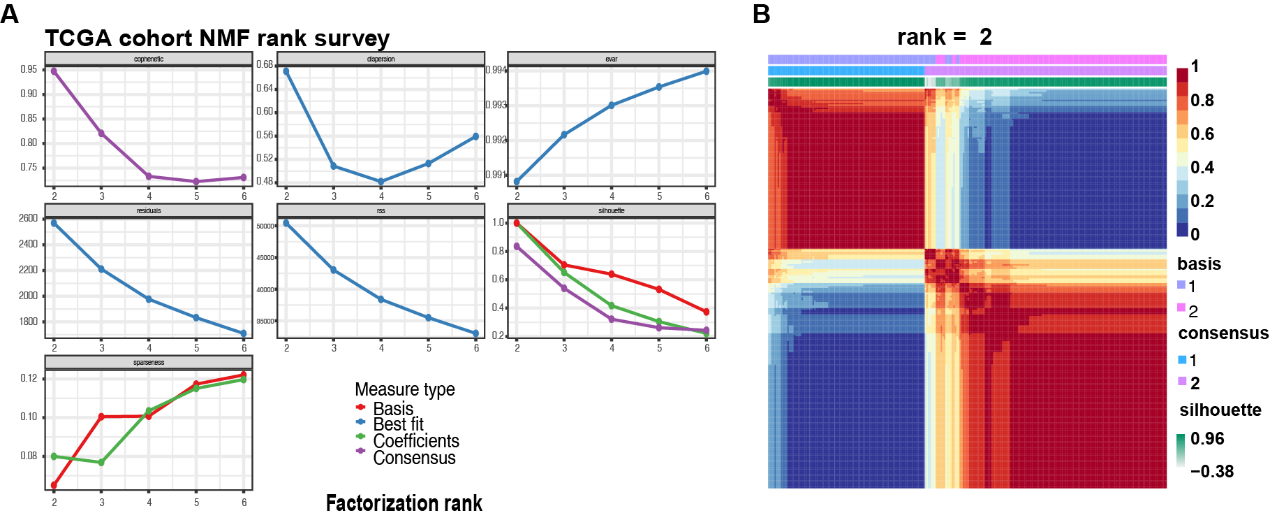


**Figure S2. (A)** The relationship between cophenetic, dispersion and silhouette coefficients with respect to 2 to 6 clusters in TCGA cohort. **(B)** Non-negative matrix factorization clustering map of rank 2 in TCGA cohort.

**Figure S3**：

**
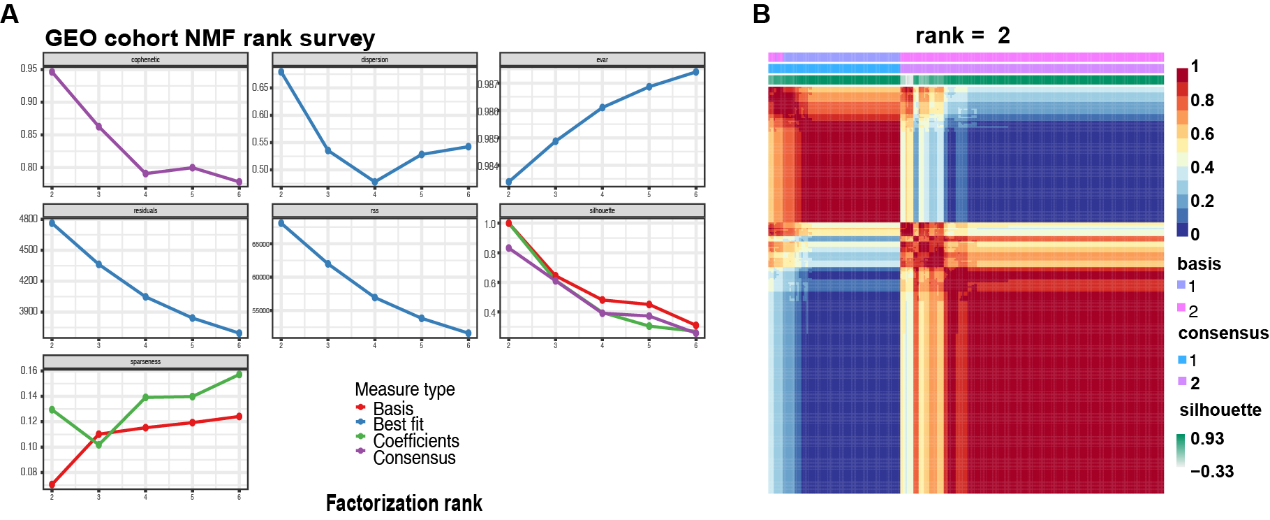
**

**Figure S3. (A)** The relationship between cophenetic, dispersion and silhouette coefficients with respect to 2 to 6 clusters in GEO cohort. **(B)** Non-negative matrix factorization clustering map of rank 2 in GEO cohort.

**Figure S4**：

| 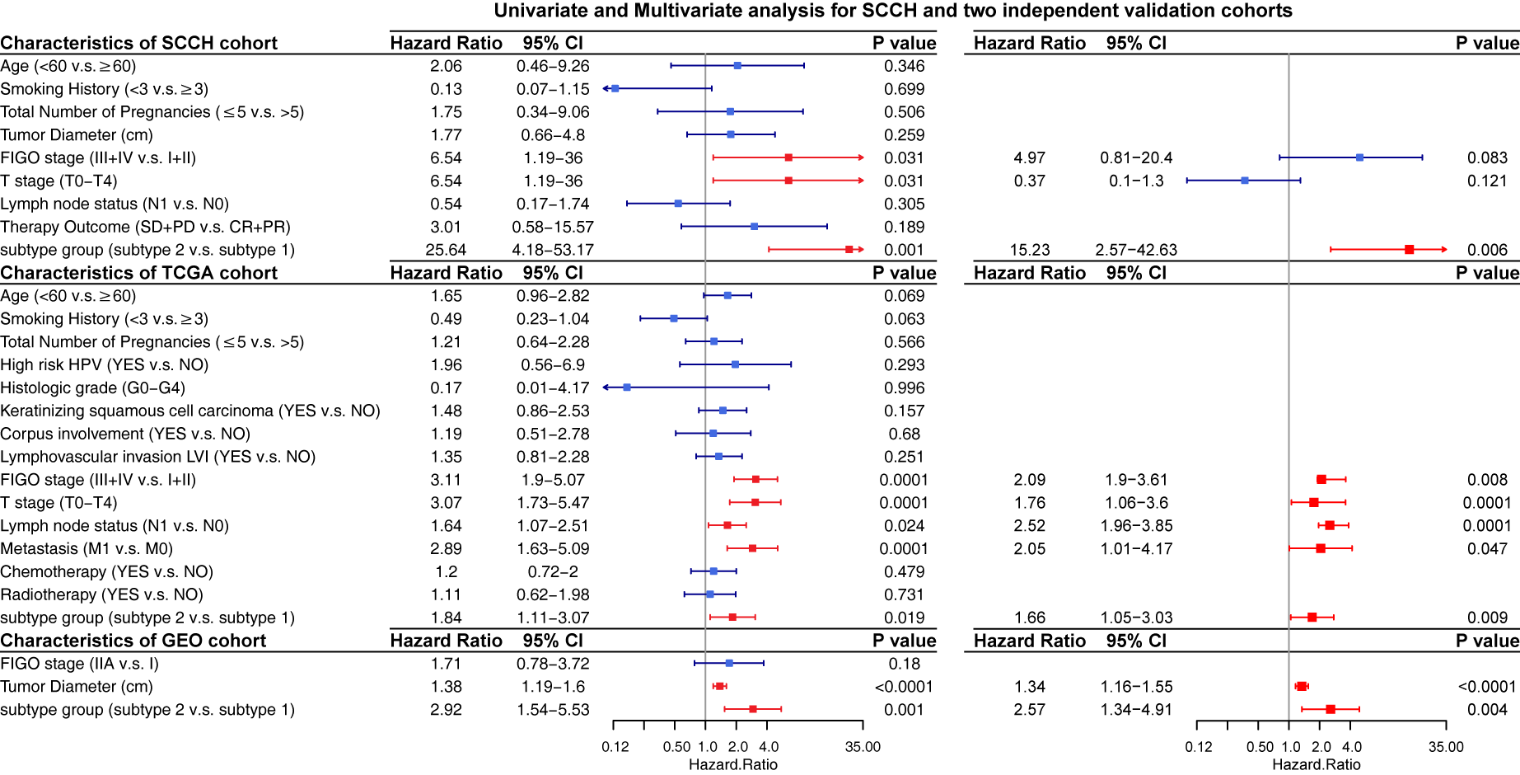  **Figure S4.** Forest plot of hazard ratios (HR) for prognostic value assessed by the cervical SCC subtype classifier and clinicopathological characteristics in the training set and validation set. Error bars represent 95% confidence intervals. The vertical grey line represents HR = 1. |
| --- |

**Figure S5**：

**
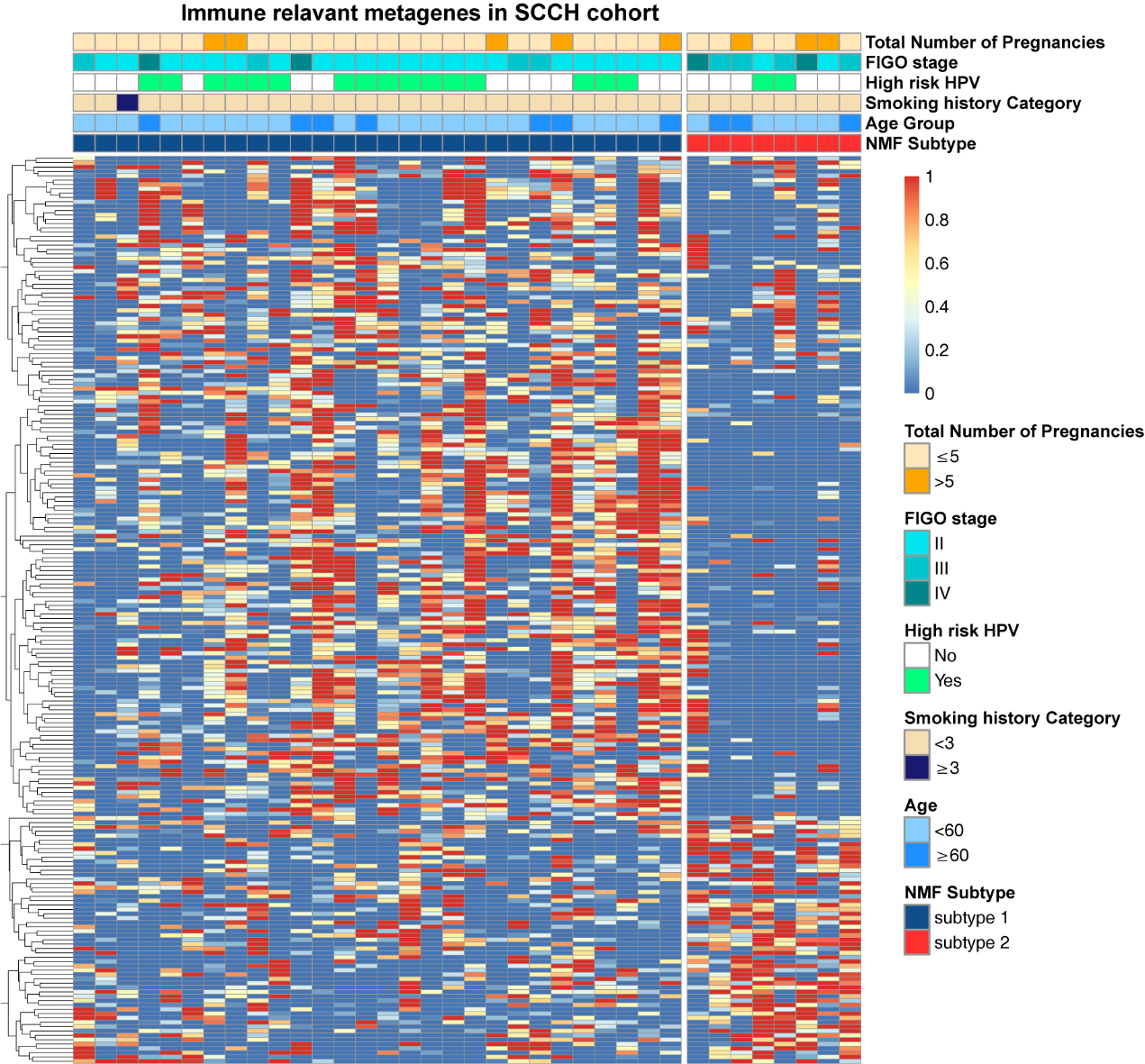
**

**Figure S5.** The distribution of clinicopathological characteristics and the different expression patterns of 257 metagenes among the two subtypes in the SCCH cohort.

**Figure S6**：


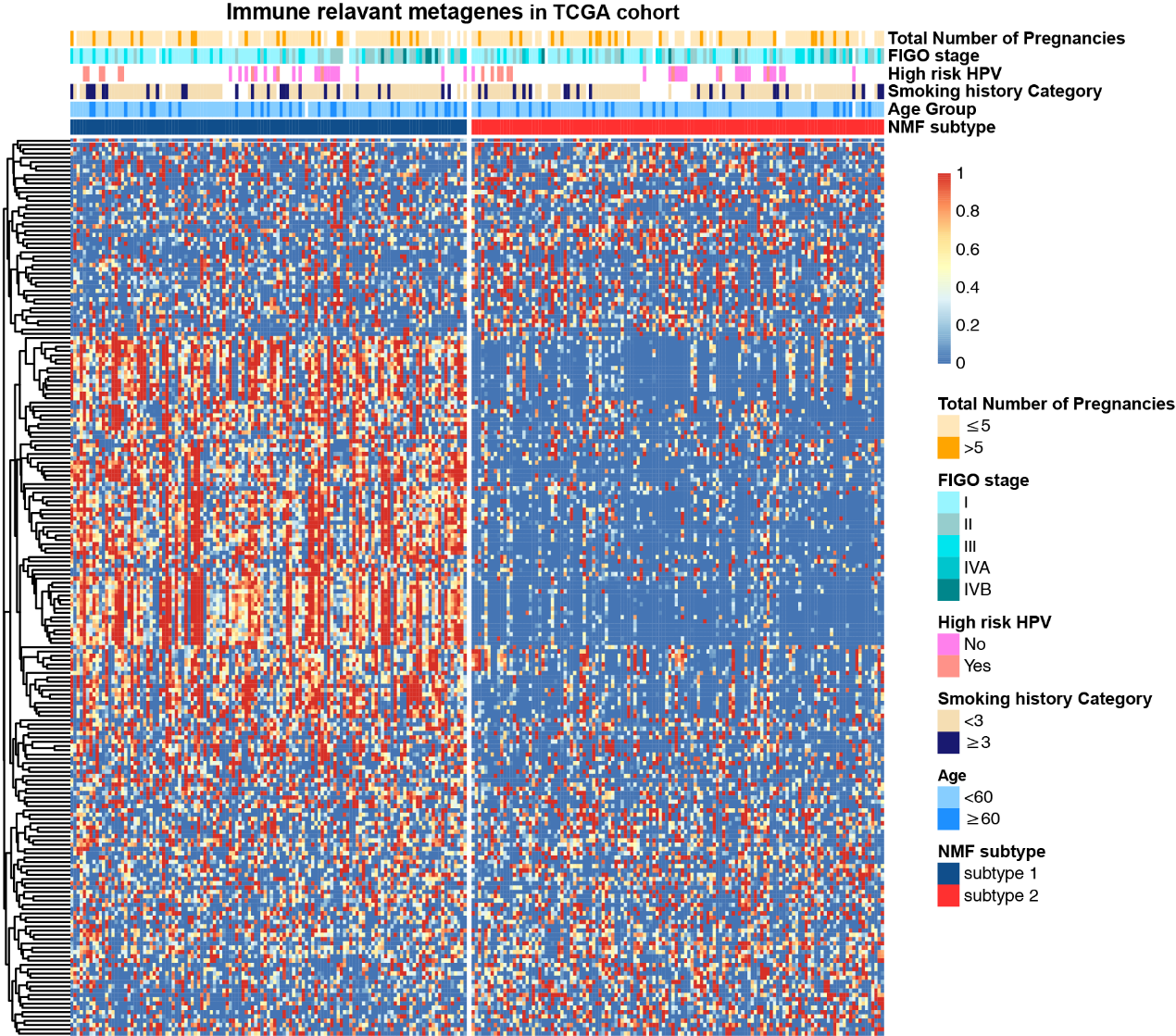


**Figure S6.** The distribution of clinicopathological characteristics and the different expression patterns of metagenes among the two subtypes in the TCGA cohort.

**Figure S7**：


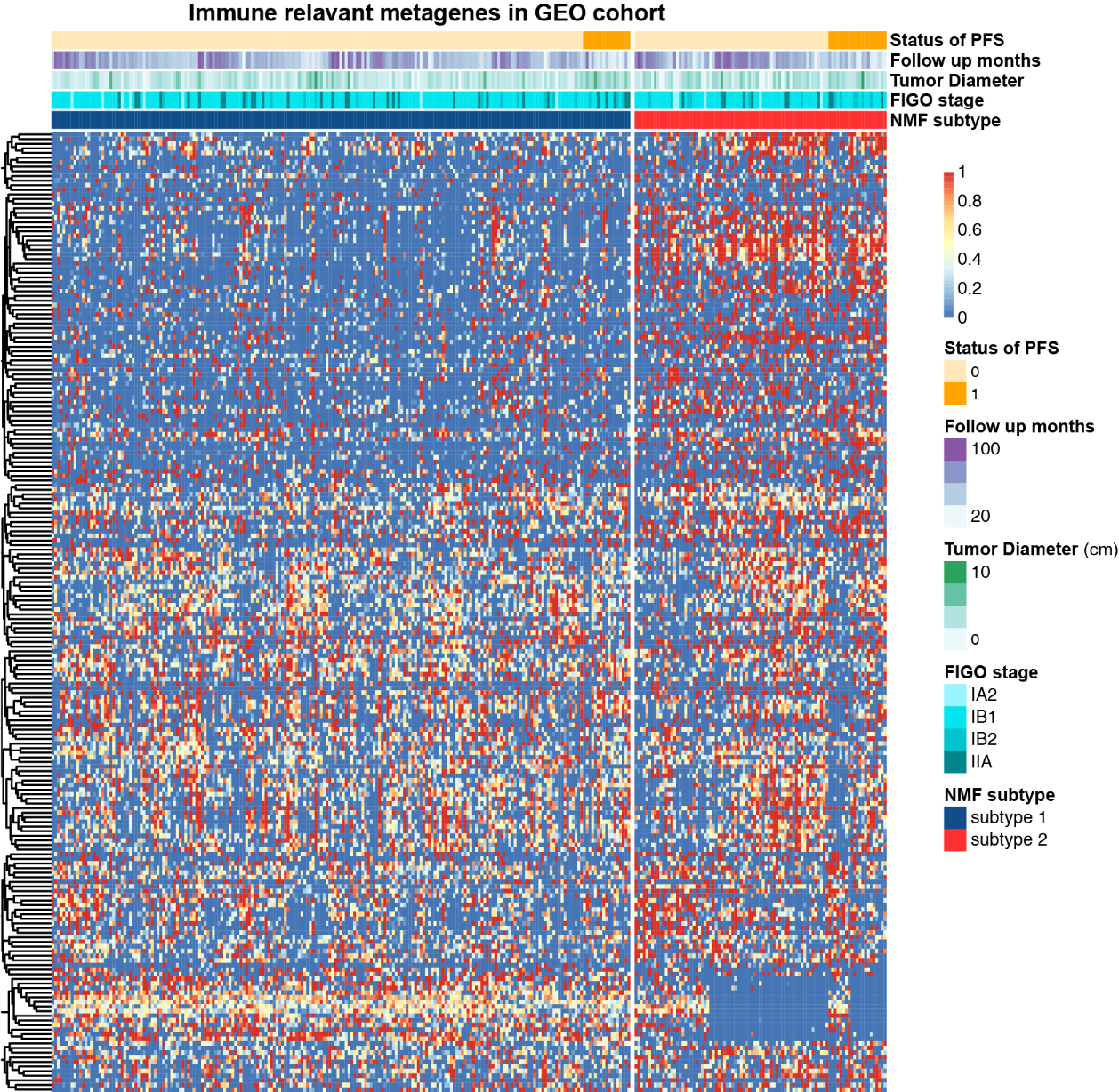


**Figure S7.** The distribution of clinicopathological characteristics and the different expression patterns of metagenes among the two subtypes in the GEO cohort.

**Figure S8**：


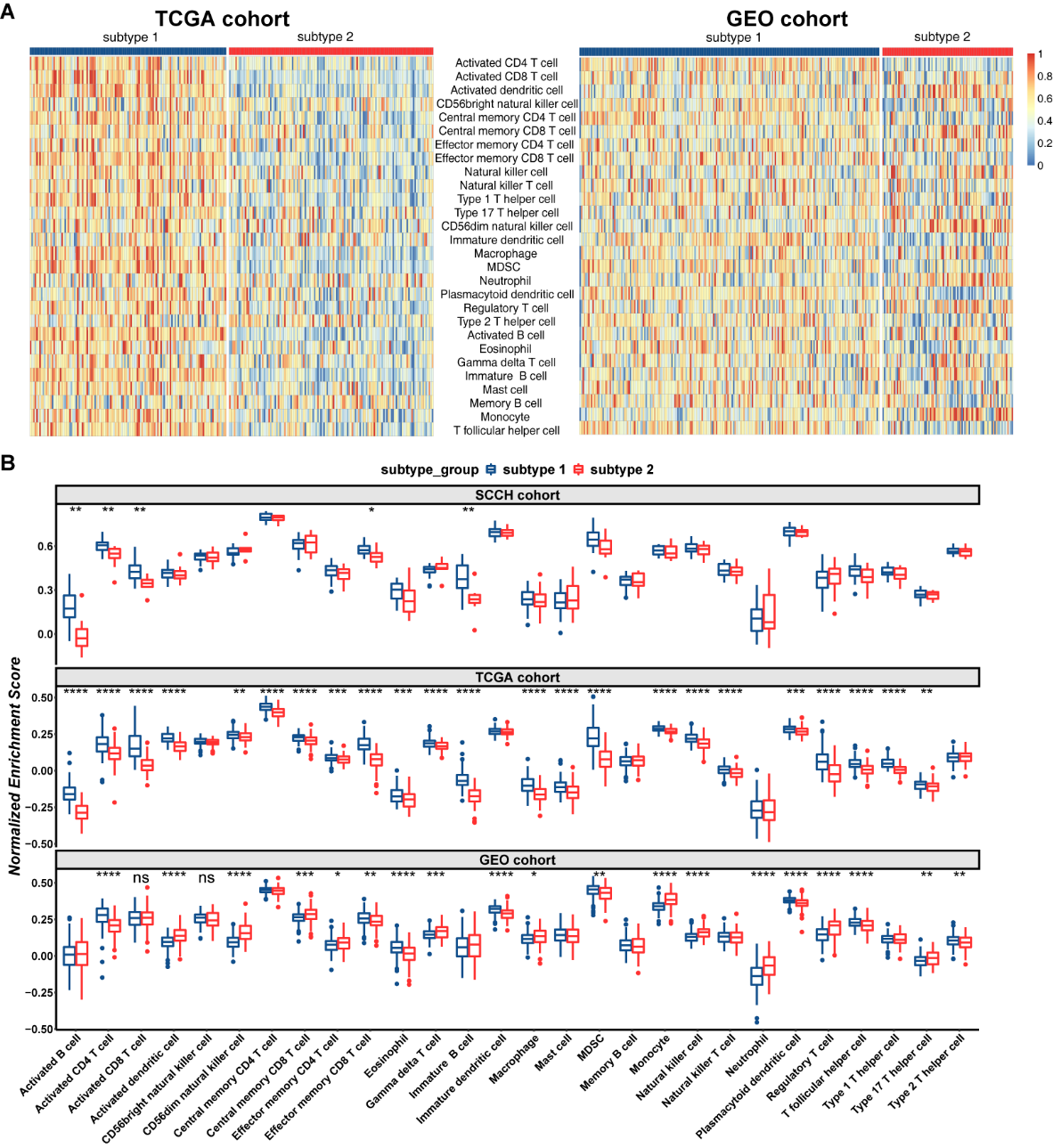


**Figure S8.** The abundance of 28 immune cell types estimated by ssGSEA algorithm between subtype 1 and subtype 2 in the **(A)** TCGA and **(B)** GEO cohort. **(C)** Box plots depicting the distribution of immune cell types among the two subtypes in all three cohorts. The normallized enrichment score (NES) was compared through the Wilcoxon rank-sum test. (* p-value < 0.05, ** P≤0.01, *** p-value≤0.001, **** P≤0.0001).

**Figure S9:**

| 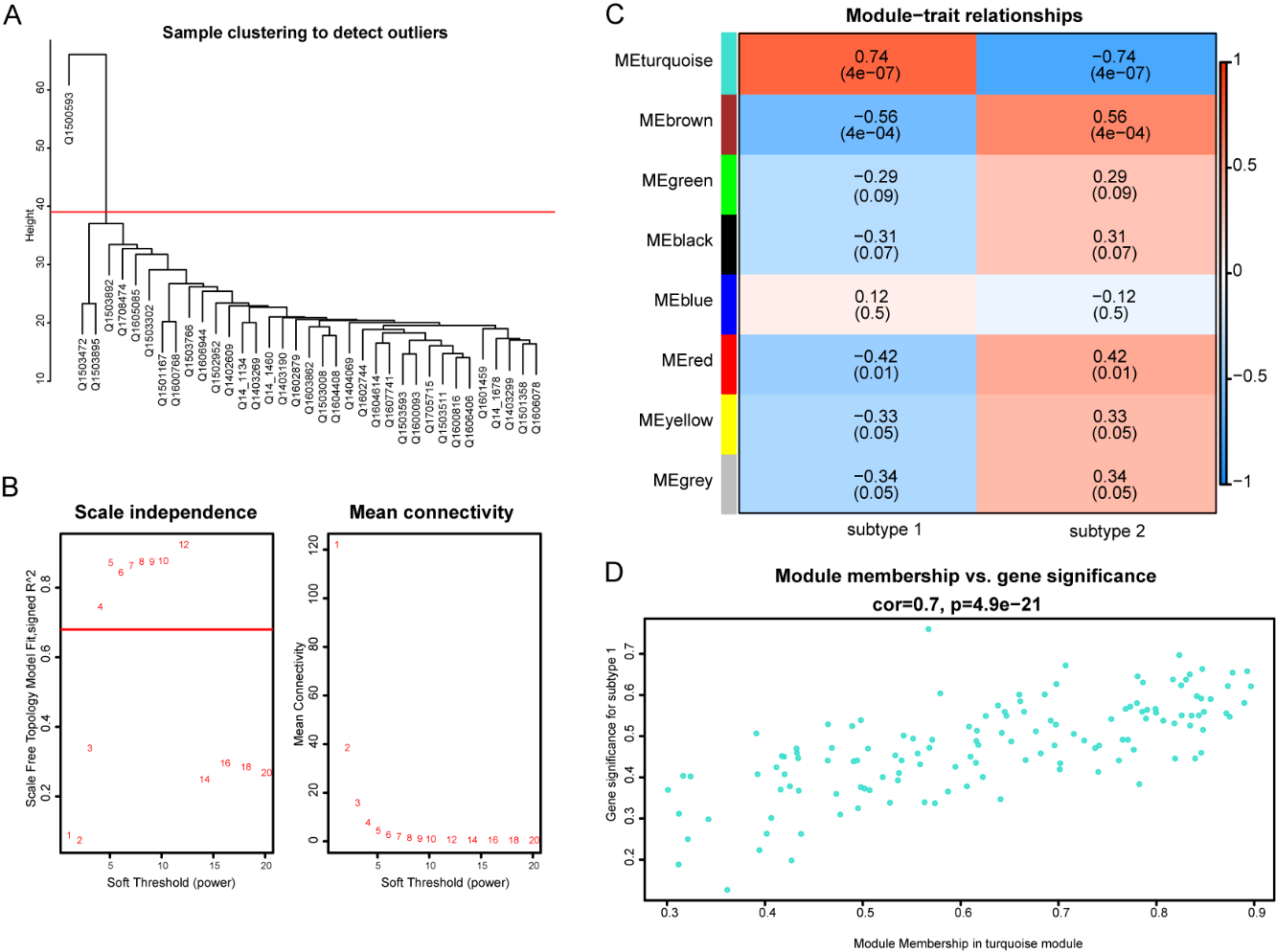  **Figure S9.** The weighted gene co-expression network analysis in SCCH cohort. **(A)** One sample was deleted as outlier after the hierarchical clustering analysis. **(B)** The power of β = 4 was selected as the optimal soft threshold. **(C)** Identification of the correlation between module eigengenes and subtypes of cervical SCC. The corresponding correlation and P-value are at the top and bottom of each cell respectively. **(D)** Correlations between the gene significance and module membership in the turquoise module. |
| --- |

**Figure S10:**

| 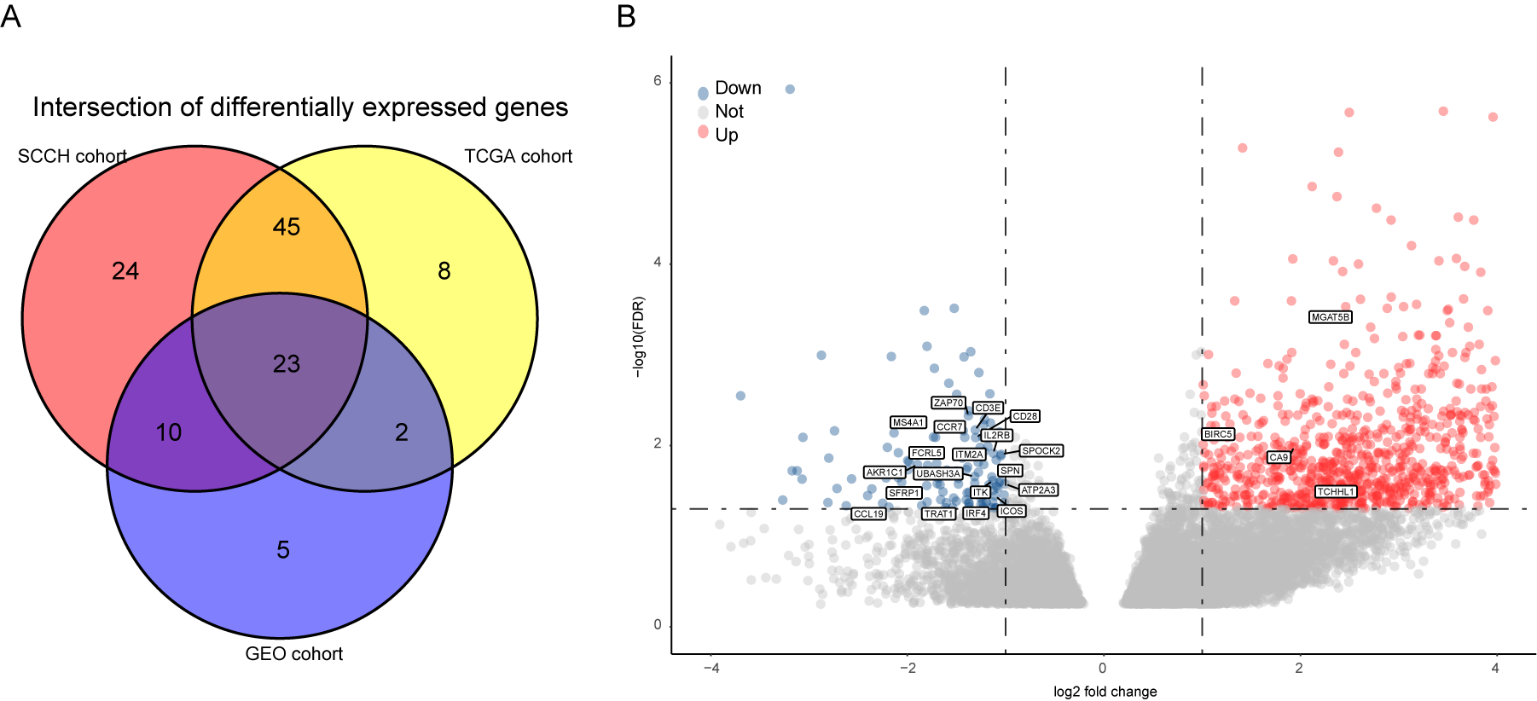  **Figure S10.** A total of 23 overlapping DEGs in the three cohorts were found. **(A)** Venn diagram illustrating the number of overlapping DEGs. **(B)** Distribution of 23 DEGs in the Volcano plots. |
| --- |

**Figure S11:**

| 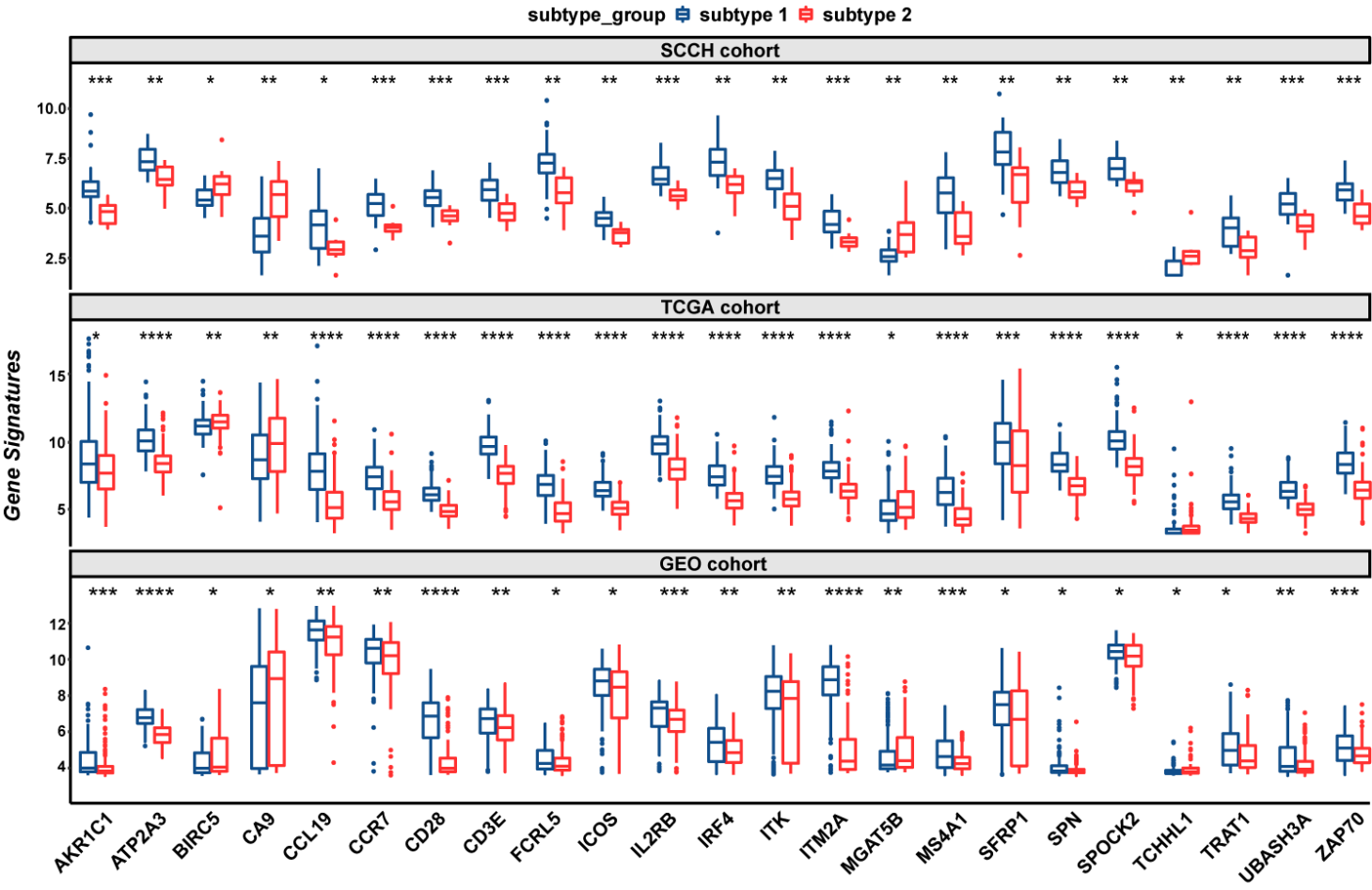  **Figure S11.** Box plots depicting the distribution of 23 overlapping DEGs among the two subtypes in all three cohorts. The expression count of DEGs was compared through the Wilcoxon rank-sum test. (* p-value < 0.05, ** P≤0.01, *** p-value≤0.001, **** P≤0.0001). |
| --- |

**Table S1：**Baseline clinical features for the cervical SCC patients in the training set and validation set.

**Table S2:** The list of immune-related genes (IRGs)

**Table S3:** The enriched 21 Kyoto Encyclopedia of Genes and Genomes (KEGG) pathways between sub1 and sub2.

**Table S4:** The enriched 138 Gene Ontology (GO) biological pathways between sub1 and sub2.

**Table S5:** The mutation frequencies of 45 driver genes of cervical SCC between sub1 and sub2.

**Table S6:** 83 mRNAs significantly related to OS used to construct the random forest classifier.

**Table S7:** 23-mRNA signature related to cervical SCC subtypes.
